# EHMTI-0096. Efficacy of sumatriptan: assessment of a possible biomarker

**DOI:** 10.1186/1129-2377-15-S1-G16

**Published:** 2014-09-18

**Authors:** K Ibrahimi, AH Danser, AH van den Meiracker, A MaassenVanDenBrink

**Affiliations:** 1 Department of Internal Medicine, Erasmus MC, Rotterdam, Netherlands

## Introduction

Sumatriptan is a frequently applied anti-migraine treatment, yet ≈20-30% migraineurs are nonresponders. TRPV1 channels on trigeminal nerve endings release CGRP during migraine attacks, while, supposedly, stimulation of the presynaptic 5-HT(1B)/1D receptor by sumatriptan inhibits this release. Capsaicin (CAP) stimulates TRPV1 channels, causing CGRP-dependent vasodilatation, whereas electrical stimulation (ES) induces vasodilation without direct TRPV1 activation.

## Aim

To assess a possible biomarker for the efficacy of sumatriptan.

## Methods

We investigated the effect of sumatriptan on the rise of dermal blood flow (DBF) of the forehead skin (innervated by the trigeminal nerve) by CAP application (0.6 mg/ml) and ES (0.2-1.0 mA) before and after subcutaneous placebo and sumatriptan in a randomized, double-blind, placebo controlled cross-over study, including healthy male (n=11, age±SD: 29±8 yrs) and female (n=11, 32±7 yrs) subjects.

## Results

DBF responses to CAP (mean±SEM: 313±16 A.U.) were significantly attenuated after sumatriptan (mean decrease DBF: 82±18 A.U., p < 0.001) but not after placebo (mean decrease DBF: 21±12 A.U., p = 0.1026), whereas DBF responses to ES were not affected by sumatriptan or placebo. Sumatriptan, but not placebo, increased blood pressure by 6±2/11±2 mmHg, p < 0.001. In 23% of the subjects, sumatriptan did not attenuate the DBF response.

## Conclusions

Sumatriptan may inhibit the release of CGRP via the stimulation of the presynaptic 5-HT(1B)/1D receptor and/or by a direct effect on TRPV1 channels. ES appears to be a nonspecific stimulus, most likely releasing other neuropeptides besides CGRP. Future studies should indicate whether nonresponse in our model correlates with clinical nonresponse to sumatriptan.

No conflict of interest.

